# DOME/GALT type adenocarcimoma of the colon: a case report, literature review and a unified phenotypic categorization

**DOI:** 10.1186/s13000-015-0305-1

**Published:** 2015-07-09

**Authors:** Hala Kannuna, Carlos A. Rubio, Patricia Caseiro Silverio, Marc Girardin, Nicolas Goossens, Laura Rubbia-Brandt, Giacomo Puppa

**Affiliations:** Department of Clinical Pathology, Geneva University Hospital, 1 rue Michel-Servet, 1211 Geneva, Switzerland; Department of Pathology, Karolinska University Hospital, Stockholm, Sweden; Division of Gastroenterology and Hepatology, Geneva University Hospital, Geneva, Switzerland

**Keywords:** Dome, GALT, Adenocarcinoma, Colon

## Abstract

Several types of colorectal cancers are associated with a prominent lymphoid component, which is considered a positive prognostic factor.

We report a case of a dome-type carcinoma of the cecum in a 57 year old female.

The sessile, non-polypoid lesion histologically consisted of a tubulovillous adenoma with low-grade dysplasia.

The submucosal invasive component showed low-grade architectural features that included cystically dilated glands containing eosinohilic debris. Immunohistochemical studies displayed retention of the four mistmach repair proteins, consistent with a stable phenotype. After 3 years, the patient remains free of recurrence.

A literature review highlighted striking similarities between dome-type carcinoma and the gut-associated lymphoid tissue carcinoma, the two sharing an intimate association with the gut associated lymphoid tissue.

The two variants might therefore be grouped into a unified category.

## Background

The term “dome-type carcinoma” (DC) was first applied by De Petris in 1999 when describing the macroscopic appearance of a dome-shaped elevation of the mucosa of a colon carcinoma associated with gut associated lymphoid tissue [1].

Since then 11 cases of this distinct variant have been reported [2–9]. There are several types of colorectal cancer associated with a dense lymphoid component, such as medullary carcinoma, lymphoepithelioma-like carcinoma (LELC) [10], gut-associated lymphoid tissue carcinoma (GALT carcinoma) [11, 12], colorectal cancer (CRC) occurring in the context of Lynch syndrome and sporadic microsatellite instability-high (MSI-H) CRC [13].

We report an additional case of so-called DC of the colon along with a literature review.

The histological and immunohistochemical diagnostic features are presented as well the differential diagnosis with similar variants.

### Case report

**Fig. 1:**
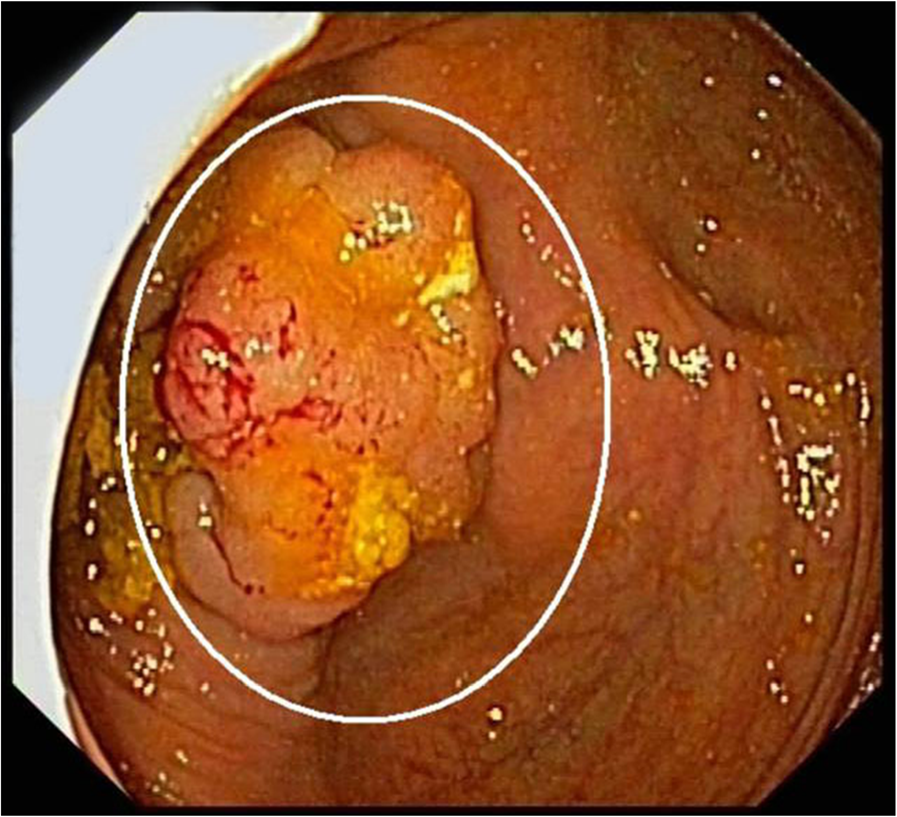
Colonoscopic image of the caecal mass with a villous aspect

A 57 year old female with no family history of CRC was hospitalized because of fever and abdominal pain. The abdominal computed tomography detected a cecal mass and colonoscopy showed two lesions: a 30 mm sessile mass in the cecum (Fig. 1) and a 40 mm pedunculated polyp in the rectum. Biopsy histology of the lesions in the right and left colon showed intramucosal adenocarcinoma and high-grade dysplastic tubulovillous adenoma respectively. The subsequent treatment consisted of an endoscopic resection of the rectal polyp and a right hemicoloectomy.

At definitive histology the rectal polyp was a tubulovillous adenoma with high-grade dysplasia.

**Fig. 2:**
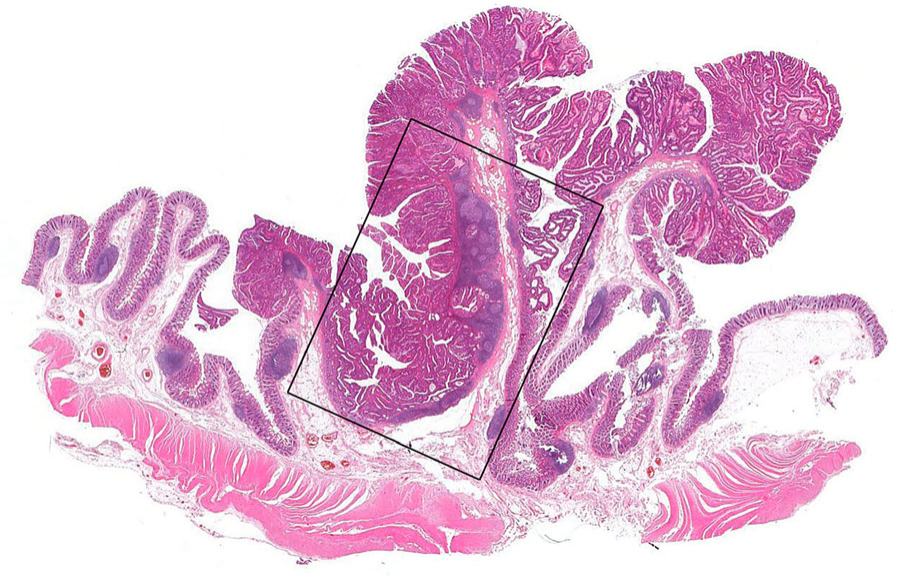
Panoramic view of the polyp (H&E; ×1). The area in the box is shown at higher-power magnification in Fig. 3

**Fig. 3:**
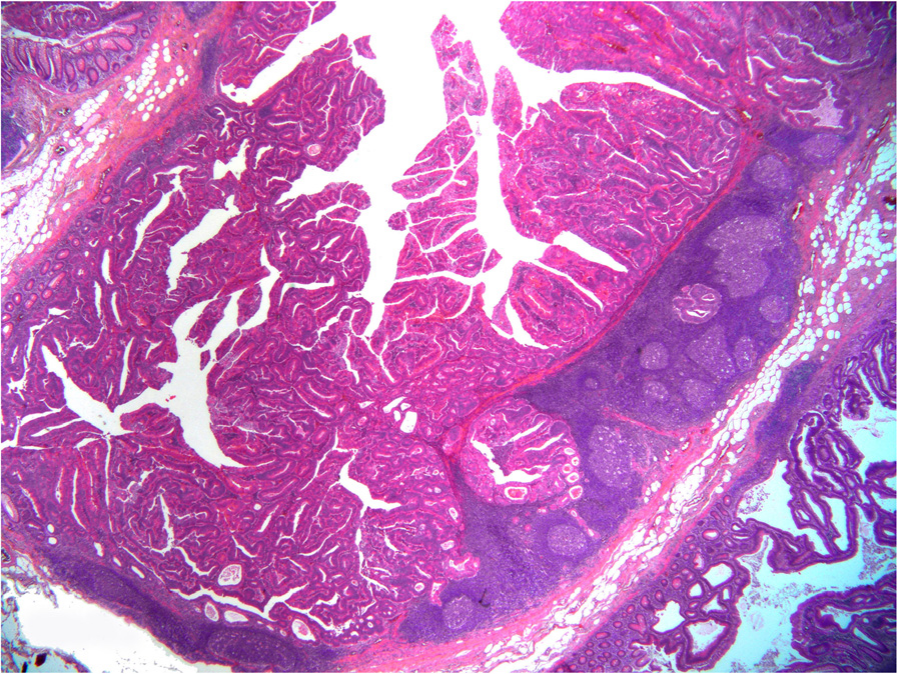
The submucosal invasive component in association with GALT (H&E; ×10)

The cecal mass was a moderately differentiated adenocarcinoma evolving from a tubulovillous adenoma, invading the submucosa (Figs. 2 and 3).

**Fig. 4:**
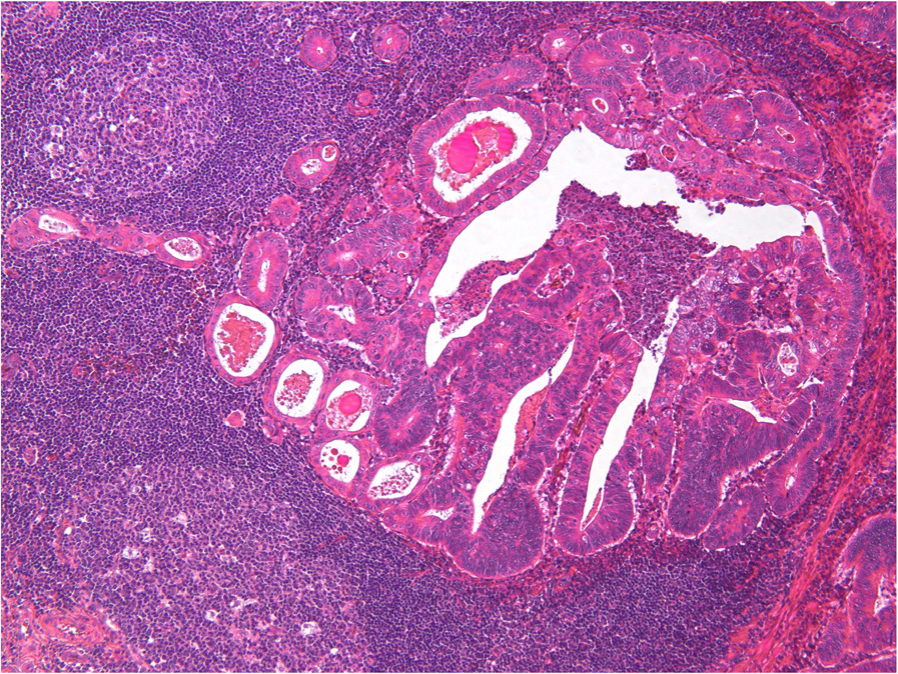
Adenocarcinoma with low-grade architectural features including cystically dilated glands with eosinohilic debris. Tumor cell cytoplasmic eosinophilia is also evident (H&E; ×50)

The submucosal tumour was surrounded by a prominent lymphoid tissue exhibiting reactive germinal centres (Figs. 3 and 4). The glands were cystically dilated with intraluminal eosinophilic debris (Fig. 4). A clear space was evident separating the glandular epithelium from the intraglandular material. Neoplastic cells lining the glands were single-layered, cuboidal to columnar, with cytoplasmic eosinophilia and moderate atypia. No desmoplasia, tumor infiltrating lymphocytes or goblet cells were observed. Eleven lymph nodes recovered, all of which were negatives for metastasis.

Immumohistochemistry showed retained expression of the mismatch repair proteins MSH2, MSH6, MLH1 and PMS2.

The patient remains recurrence free after three years of follow-up.

### Literature review

Since the original report in late 90’s by De Petris [1], 11 cases of DC has been reported [2–9].

All of these cases were presented matching the macroscopic and histopathological features as described by De Petris: The macroscopic dome-like, non polypoid appearance and the architecture encompassing dilated malignant glands lined by columnar epithelium with eosinophilic cytoplasm on a prominent lymphoid background [1].

The earliest report of carcinoma of the colon originating in lymphoid-associated mucosa was in 1984, describing the lesion in a patient with ulcerative colitis [14]. A few additional cases of GALT carcinoma have since been reported [11, 12, 15].

Tables 1 and 2 present the clinico-pathological features of the DC and GALT carcinomas.

**Table 1:** Clinico- pathological characteristics of the reported cases of Dome type and GALT carcinoma of the colon

Author, Year	Age, Sex	Presentation	Family history	Location	Macroscopic aspect	Staging	Mismatch repair status
			CRC				
1) Rubio, 1984	NM	Surveillance for UC	NM	NM	NM	NM	NM
2) De Petris, 1999	44, M	Pain, weight loss, anemia	Lynch S.	Ascending colon	9-mm dome-shaped lesion	pT1 N0	Not performed
3) Jass, 2000	56, M	Screening for FAP	FAP (daughter)	Ascending colon	30-mm plaque	pT1 N0	Stable (PCR)
4) Clouston, 2000	63, F	Not stated	NM	Sigmoid	8-mm sessile polyp	pT1 N0	NM
5) Clouston, 2000	56, M	Not stated	NM	Sigmoid	14-mm sessile polyp	pT1 N0	NM
6) Rubio, 2002	53, F	Surveillance for UC	NM	Ascending colon	sessile polyp	pT1 N0	Not performed
7) Asmussen, 2008	76, F	Rectal bleeding	NM	Sigmoid	20-mm plaque	pT1 N0	Stable (IHC)
8) Asmussen, 2008	86, F	Rectal bleeding	NM	Anorectal	24-mm plaque	pT2 N0	Stable (IHC)
9) Stewart, 2008	70, M	Surveillance for UC	No	Ascending colon	5-mm polyp/raised area	pT1 N0	Stable (IHC)
10) Stewart, 2008	63, F	Diverticulitis	No	Transverse colon	17-mm plaque	pT1 N0	Stable (IHC)
11) Rubio, 2010	53, F	Screening (Lynch syndrome)	Yes	Ascending colon	8 mm plaque	pT1 N0	Instable (IHC)
12) Coyne, 2011	76, M	Screening	Yes	Caecum	23-mm ulcerated plaque	pT1 N0	Stable (IHC)
13) Yamada, 2012	77, M	Abdominal discomfort	NM	Transverse colon	30-mm SMT-like lesion	pT3 N0	Stable (IHC)
14) Puppa, 2012	56, M	Painful constipation	No	Right flexure	8-mm plaque	pT1 N0	Stable (IHC)
15) Yamada, 2013	76, F	Treatment of rectal SMT	NM	Lower rectum	10-mm SMT-like lesion	pT1 N0	Stable (IHC)
16) Rubio, 2013	68, F	Surveillance for UC	NM	Transverse colon	NM	pT1 N0	Not performed
17) Current case	57, F	Fever, abdominal pain	No	Caecum	30 mm sessile polyp	pT1 N0	Stable (IHC)

*NM*: not mentionned; *UC*: ulcerative colitis; *FAP* : Familial adenomatous polyposis; *SMT*: submucosal tumor; *IHC*: immunohistochemistry

**Table 2:** Microscopic features of Dome and GALT carcinoma of the colon

Author, Year	Associatedadenoma	Adenoc. grade	Associated usual-type adenoc.	Lymphoid stroma, reactive germinal centers	Architecture dilated cystic glands luminal pink material	Intraglandular necrosis	Cytological features: columnar cells, no goblet cells, mild atypia, no desmoplasia	TIL	Used name
1) Rubio, 1984	Absent	LG	No	NM	NM	NM	NM	NM	GC
2) De Petris, 1999	Absent	LG	No	Present	Yes	Present	Yes	Absent	DC
3) Jass, 2000	Absent	LG	No	Present	Yes	Present	Yes	Present	DC
4) Clouston, 2000	Present	LG	Yes	Present	Yes	Present	Yes	Present	DC
5) Clouston, 2000	Absent	LG	No	Present	Yes	Present	Yes	Absent	DC
6) Rubio, 2002	HGD	LG	No	Present	Yes	Present	Yes	Absent	GC
7) Asmussen, 2008	Absent	LG	No	Present	Yes	Absent	Yes	Present	DC
8) Asmussen, 2008	Absent	LG	Yes	Present	Yes	Absent	Yes	Absent	DC
9) Stewart, 2008	Absent	LG	No	Present	Yes	NM	Yes	Present	DC
10) Stewart, 2008	Absent	LG	No	Present	Yes	NM	Yes	Absent	DC
11) Rubio, 2010	Absent	LG	No	Present	NM	Absent	yes	Present	GC/DC
12) Coyne, 2011	HGD	LG	Yes	Present	Yes	Present	Yes	Absent	DC
13) Yamada, 2012	HGD	LG	No	Present	Yes	Present	Yes	NM	DC
14) Puppa, 2012	HGD	LG	No	Present	Yes	Absent	Yes	Absent	DC
15) Yamada, 2013	Absent	LG	No	Present	Yes	NM	Yes	NM	DC
16) Rubio, 2013	HGD	LG	No	Present	No	Absent	Yes	Absent	GC
17) Current case	HGD	LG	No	Present	Yes	Present	Yes	Absent	DC

Adenoc: adenocarcinoma; *HGD*: High grade dysplasia; *LLC*: Lymphoepithelioma-like carcinoma *DC*: dome-type adenocarcinoma; *GC*: GALT carcinoma; *LG*: low grade; *NM*: not mentionned

As with DC, GALT-carcinoma has a plaque/sessile macroscopic appearance; it is limited to the submucosa, is associated with GALT and shows a low differentiation grade.

Clinical presentations of DC and GALT-carcinoma are reported either as sporadic-type colon cancer or in association with ulcerative colitis, familial adenomatous polyposis, Lynch syndrome and other positive family histories of colorectal cancer, in both right and left colon (Table 1) therefore it seems that the two tumor types are not associated with any specific mechanisms of tumor predisposition [6].

Another similarity is that almost all tumors reported are early-invasive, limited to the submucosa (T1) except in cases of DC reported by Amussen in 2008 (T2) [2] and by Yamada in 2012 (T3) [8]. No metastases to lymph nodes or to distant sites are reported.

On immunohistochemistry, all DCs studied for mismatch repair protein expression showed retention of all 4 proteins. One case of GALT-carcinoma arose in a patient with Lynch syndrome and accordingly showed microsatellite instability [11].

Despite an overlap in key histological features between DC and GALT-carcinoma, there is variability in some ancillary aspects including tumor infiltrating lymphocytes, intraacinar necrosis, the presence of a preexisting adenoma remnant and foci of usual-type adenocarcinoma.

## Discussion

The mucosal-associated lymphoid tissue (MALT) in the intestine is termed gut-associated lymphoid tissue (GALT). It consists of isolated and aggregated lymphoid follicles [16].

The discrete lymphoid aggregates form dome-like masses that bulge into the gut lumen [5].

A follicle-associated epithelium (FAE) overlies the aggregated lymphoid follicles. Such epithelium is a single cell layer composed of enterocytes and specialized epithelial microfold cells, so called “M-cells”, and is devoid of goblet and enteroendocrine cells [16].

A very small minority of CRC is thought to derive from M-cell. In 1999, De Petris described the dome-shaped elevation of the mucosa corresponding to a small submucosal adenocarcinoma composed of dilated malignant glands lined by columnar epithelium with eosinophilic cytoplasm and hyperchromatic nuclei on a prominent lymphoid background [1].

De Petris speculated that that DC is the malignant counterpart of the lymphoglandular complex and it may represent a precursor of LELC in view of the tumor-associated lymphoid stroma and the presence of less well differentiated areas [1].

There are three arguments for a linkage between the lymphoglandular complex and DC thus supporting the concept that this tumor may originate from M-cells of the FAE.

Firstly the malignant epithelium of such a tumor present an intimate relationship with a conspicuous lymphoid tissue showing the characteristic organization of GALT [5].

Secondly, there is the observation that DC, as in FAE, is described lacking goblet cells [2].

Finally, there are morphological similarities between the neoplastic changes observed in rat experimental models and the morphology of DC observed in reported human cases [2].

In this regard, studies of colon tumors in experimental carcinogen-treated rats showed a significant association between the location of lymphoid aggregates, the location of sessile-type non polypoid tumors and an early appearance of malignant glands inside the aggregates [17, 18].

Regarding histological and diagnostic features, DC show a constellation of morphological features other than the prominent lymphoid component which are variably present (Table 2), thus accounting for some heterogeneity.

The presence of tumor infiltrating lymphocytes is reported in half of the cases of DC (Table 2).

Despite M-cells normally exhibit numerous intercellular lymphocytes, this may not always the case: accordingly the amount of lymphocytes is related to the state of maturity of these cells [2, 19].

A preexisting associated adenoma has been described in almost half of both DC and GALT carcinoma (Table 2).

An explanation for the absence of an adenomatous component could be that some of these tumors arise from submucosal FAE or from herniated glands in the submucosa, typically in UC, as described by one of us [12, 14].

When present, an adenomatous transformation may in turn be induced by the lymphoid follicles themselves in the colonic mucosa/submucosa [20].

The presence of usual type adenocarcinoma observed within some DC could explain the relative rarity of DC as an entity, by which the DC component is obliterated by overgrowth of the usual type carcinoma [3].

The only difference among the DC and the GALT-carcinoma is the unique cyto-architectural features described by De Petris. Evidence of the cystically dilated glands and the cytological features typical of DC are reported in only one case of GALT-carcinoma by Rubio in 2002 [15].

Medullary carcinoma and lymphoepithelioma-like carcinoma differs from DC and GALT-carcinoma in several ways. In cyto-architectural features, they show high-grade-undifferentiated features [10] and they lack the typical DC cytological features.

The medullary phenotype is associated with MSI-H CRC [10] and accordingly this variant shows pushing margins and striking peritumoral lymphoid infiltrates besides intratumoral [1].

The most important morphological features serving as diagnostically useful markers of MSI-H-CRC occurring sporadically and in the context of Lynch syndrome are lymphocytic infiltration, mucin secretion and poor differentiation [13].

Patterns of lymphocytic infiltration in MSI-H CRC include a nodular or Crohn’s-like peritumoural lymphocytic reaction and the presence of tumour infiltrating lymphocytes (TILs) [13].

The stromal lymphoid component associated with DC and GALT carcinoma organized with lymphoid follicles and germinal centres differs from the lymphoid infiltrates encountered in medullary carcinoma, lymphoepithelioma-like carcinoma and in MSI-H CRC; most probably it represent remnants of pre-existing lymphoid nodules rather than an adaptive immune response [2], thus accounting for histological heterogeneity and the difference in microsatellite status.

Since any kind of lymphocytic infiltration (peritumoral inflammatory reaction, TILs and Crohn-like reaction) in CRC is considered a positive prognostic factor [21], it is not surprising that there are no reported recurrences nor cancer-associated deaths from DC or GALT-carcinoma.

## Conclusions

We report an additional case of so-called DC of the colon showing typical staging, morphological and immunohistochemical features: An early and low grade lesion, associated with a conspicuous lymphoid tissue showing the characteristic organization of GALT, lacking features of biological aggressiveness and with retained expression of 4 mismatch repair proteins.

The association of morphological features, in particular the pattern of lymphocytic infiltration with the immumohistochemical mismatch repair status allows the distinction from other types of CRC associated with a prominent lymphoid component.

We highlighted the similarities between DC and the GALT-carcinoma, the two showing an intimate relationship with lymphoid tissue with the characteristic organization of GALT.

Despite heterogeneity in tumour presentation (arising in the context of both sporadic and syndromic cases), and some variable ancillary morphological features both DC and GALT-carcinoma could be grouped in a single category.

Since the term “dome” is an endoscopic descriptor lacking in diagnostic histopathological specificity we consider the term GALT carcinoma more appropriate for categorization purposes.

### Consent

Written informed consent was obtained from the patient for publication of this Case Report and any accompanying images.
